# Altered glucose profiles and risk for hypoglycaemia during oral glucose tolerance testing in pregnancies after gastric bypass surgery

**DOI:** 10.1007/s00125-016-4128-8

**Published:** 2016-10-18

**Authors:** Michael Feichtinger, Tina Stopp, Sandra Hofmann, Stephanie Springer, Sophie Pils, Alexandra Kautzky-Willer, Herbert Kiss, Wolfgang Eppel, Andrea Tura, Latife Bozkurt, Christian S. Göbl

**Affiliations:** 1 Department of Obstetrics and Gynaecology, Division of Obstetrics and Feto-maternal Medicine, Medical University of Vienna, Waehringer Guertel 18–20, A-1090 Vienna, Austria; 2 Department of Internal Medicine III, Division of Endocrinology and Metabolism, Medical University of Vienna, Vienna, Austria; 3 Metabolic Unit, Institute of Neuroscience, National Research Council, Padua, Italy

**Keywords:** Bariatric surgery, Hypoglycaemia, OGTT, Pre-eclampsia, Pregnancy

## Abstract

**Aims/hypothesis:**

A history of gastric bypass surgery can influence the results of the OGTT recommended during pregnancy. Therefore, we compared OGTT glucose kinetics and pregnancy outcome between pregnant gastric bypass patients and BMI-matched, lean and obese controls.

**Methods:**

Medical records were used to collect data on glucose measurements during the 2 h 75 g OGTT as well as on pregnancy and fetal outcome for 304 women (*n* = 76 per group, matched for age and date of delivery).

**Results:**

Women after bariatric surgery had lower fasting glucose levels compared with lean, obese and BMI-matched controls, and showed altered postprandial glucose kinetics, including a rise at 60 min followed by hypoglycaemia with serum glucose of <3.34 mmol/l (which occurred in 54.8%). Moreover, their risk of pre-eclampsia or gestational hypertension was reduced, with an increased risk of delivering small for gestational age infants.

**Conclusions/interpretation:**

Alternative strategies to accurately define impaired glucose metabolism in pregnancies after bariatric surgery should be explored.

**Electronic supplementary material:**

The online version of this article (doi:10.1007/s00125-016-4128-8) contains peer-reviewed but unedited supplementary material, which is available to authorised users.

## Introduction

The International Association of Diabetes in Pregnancy Study Groups (IADPSG) 2010 criteria proposed performing an OGTT between the 24th and 28th week of gestation as the gold standard to diagnose gestational diabetes mellitus (GDM) [1]. After adoption of these criteria by other health-care organisations, some countries (e.g. Austria) implemented the OGTT as a mandatory examination in pregnancy. However, there might be populations in which this screening strategy is less effective or even harmful.

Nowadays, owing to the worldwide rising obesity prevalence, malabsorptive surgery (such as Roux-en-Y gastric bypass) is an emerging treatment that has beneficial effects on glucose metabolism [2]. As gastric bypass surgery is associated with an altered rise in the levels of nutrients (especially glucose), it can potentially influence the results of the OGTT and therefore the transferability of diagnostic guidelines [3]. To our knowledge, the extent of altered glucose kinetics in pregnancies following bariatric surgery and its specific impact on GDM diagnosis, as well as effects on the growing fetus, have not been thoroughly investigated until now.

## Methods

### Study participants

This retrospective cohort study included a total of 304 women who attended our pregnancy out-patient clinic between January 2007 and January 2016: 76 after gastric bypass surgery (i.e. all pregnant women for whom a history of gastric bypass surgery was reported in this period), 76 with preconceptional obesity (preconceptional BMI ≥30 kg/m^2^), 76 normal weight (preconceptional BMI 18–25 kg/m^2^) and 76 BMI-matched controls. The groups were additionally matched for maternal age and date of delivery. Medical records were used to collect data on maternal variables including results of the routinely performed 2 h 75 g OGTT examination (that measures fasting and 60 min and 120 min post-load glucose levels), as well as pregnancy outcome and infant body measures at delivery. Calculations of age- and sex-adjusted percentiles of the Austrian population were based on an analysis of the local growth standard curves. Large for gestational age (LGA) and small for gestational age (SGA) were defined as a bodyweight above the 90th percentile or below the 10th percentile, respectively. The study was approved by the local ethics committee.

### Statistical analysis

Continuous and categorical variables were summarised as means ± SD (or medians [interquartile range]) and counts and percentages, respectively. Multiple comparisons (gastric bypass vs all other subgroups) were performed by ANOVA and Dunnett post hoc tests (for continuous variables) or Fisher’s exact test followed by the Bonferroni–Holm correction (for categorical variables) to achieve a 95% coverage probability.

Statistical analysis was performed with R (version 3.2.4, R Foundation for Statistical Computing, Vienna, Austria) and add-ons (multcomp, beeswarm and lattice). The two-sided significance level was set to 0.05.

## Results

**Fig. 1:**
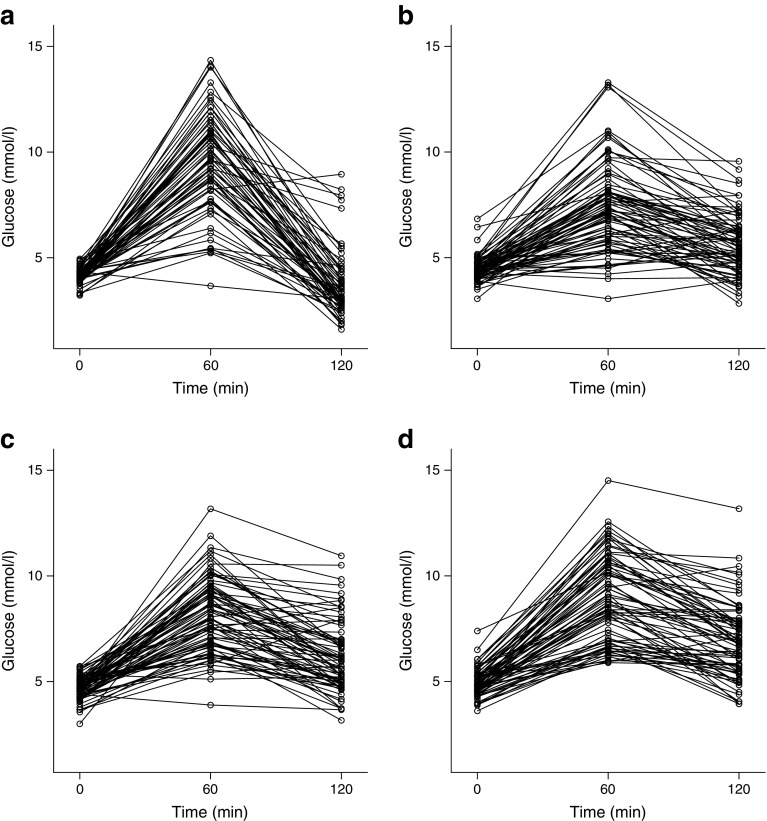
Spaghetti plots of plasma glucose kinetics during a 2 h OGTT in (**a**) pregnant patients after gastric bypass and in (**b**) normal weight, (**c**) BMI-matched and (**d**) obese controls

A description of the study population is provided in Table 1. While maternal age and parity were comparable among groups, we observed characteristic plasma glucose kinetics profiles during the OGTT. In particular, patients after bariatric surgery, who started with the lowest plasma glucose at fasting, showed a significant increase at 60 min following an oral glucose load. However, at 120 min after ingestion, glucose concentrations tended to decrease to below baseline levels in this subgroup. Postprandial hypoglycaemia (defined as a plasma glucose level of <3.34 mmol/l) was observed in 54.8% of pregnant women after gastric bypass surgery. Therefore, postprandial glucose kinetics in these patients were strikingly divergent compared with normal weight, BMI-matched and obese women; the latter group of obese women had no hypoglycaemic episodes but the highest risk of fasting and postprandial hyperglycaemia (Fig. 1). Accordingly, a diagnosis of GDM based on the IADPSG criteria was most commonly observed after 60 min in gastric bypass patients. When only fasting or 120 min glucose levels were considered, gastric bypass patients had a significantly lower incidence of GDM compared with obese and weight-matched controls. Postprandial hypoglycaemia at 120 min occurred in 39.3% of women with gastric bypass surgery, who exceeded the IADPSG thresholds at 60 min.

**Table 1:** Characteristics of the study population

Maternal/fetal characteristic	GBS		NW		BMIM		OB	
	*n*	Value	*n*	Value	*n*	Value	*n*	Value
Age, years	76	31.6 ± 6.3	76	31.5 ± 6.1	76	31.5 ± 5.5	76	31.6 ± 5.7
BMI, kg/m^2^	74	30.4 ± 5.9	76	21.8 ± 1.7*	76	30.0 ± 5.9	76	35.0 ± 4.3*
FG, mmol/l	62	4.16 ± 0.37	76	4.45 ± 0.57*	76	4.69 ± 0.52*	74	4.94 ± 0.63*
FG ≥ 5.1 mmol/l	62	0 (0.0)	76	5 (6.6)*	76	17 (22.4)*	74	26 (35.1)*
G-60, mmol/l	62	9.46 ± 2.41	76	7.42 ± 2.12*	76	8.16 ± 1.78*	74	8.93 ± 2.04
ΔG-60 (vs FG), mmol/l	62	5.30 ± 2.38	76	2.96 ± 1.95*	76	3.47 ± 1.79*	74	3.99 ± 1.78*
G-60 ≥ 10.0 mmol/l	62	27 (43.5)	76	10 (13.2)*	76	13 (17.1)*	74	27 (36.5)
G-120, mmol/l	62	3.72 ± 1.60	76	5.62 ± 1.41*	76	6.18 ± 1.68*	74	6.93 ± 1.79*
ΔG-120 (vs FG), mmol/l	62	−0.44 ± 1.55	76	1.16 ± 1.31*	76	1.49 ± 1.75*	74	2.00 ± 1.61*
G-120 ≥ 8.5 mmol/l	62	1 (1.6)	76	4 (5.3)	76	10 (13.2)*	74	14 (18.9)*
G-120 < 3.34 mmol/l	62	34 (54.8)	76	3 (3.9)*	76	1 (1.3)*	74	0 (0.0)*
GDM^b^ (overall)	62	28 (45.2)	76	12 (15.8)*	76	31 (40.8)	74	39 (52.7)
GDM^b^ (FG or G-60)	62	27 (43.5)	76	11 (14.5)*	76	27 (35.5)	74	38 (51.4)
GDM^b^ (FG or G-120)	62	1 ( 1.6)	76	7 (9.2)	76	26 (34.2)*	74	34 (45.9)*
Insulin treatment	76	11 (14.5)	76	7 (9.2)	76	16 (21.1)	76	19 (25.0)
Hypertension or PE	72	1 (1.4)	76	5 (6.6)	76	6 (7.9)	76	11 (14.5)*
Parity	76	2 (1–3)	76	2 (1–2)	76	2 (1–2.25)	76	2 (1–3)
Reproductive medicine	76	5 (6.6)	76	7 (9.2)	76	4 (5.3)	76	5 (6.6)
Multiple pregnancy	76	7 (9.2)	76	8 (10.5)	76	13 (17.1)	76	8 (10.5)
Lung maturity induction^a^	65	12 (18.5)	68	4 (5.9)	63	3 (4.8)	68	8 (11.8)
Induction of labour^a^	65	6 (9.2)	68	10 (14.7)	63	7 (11.1)	68	8 (11.8)
Caesarean section^a^	64	31 (48.4)	68	25 (36.8)	63	43 (68.3)	68	39 (57.4)
Neonatal intensive care^a^	64	7 (10.9)	68	5 (7.4)	63	5 (7.9)	68	5 (7.4)
GAD^a^, weeks	64	39 (38–40)	68	40 (39–40)	63	39 (38–39)	68	39 (38–40)
Birthweight^a^, percentile	64	32.7 ± 26.3	68	44.7 ± 29.7*	63	51.7 ± 27.5*	66	52.2 ± 28.2*
Birth length^a^, percentile	62	37.2 ± 27.9	66	47.3 ± 28.1	61	51.3 ± 27.8*	66	44.3 ± 28.0
Head^a^, percentile	62	38.4 ± 26.3	68	39.0 ± 26.5	59	50.6 ± 30.2*	61	52.4 ± 28.4*
SGA^a^	64	18 (28.1)	68	11 (16.2)	63	7 (11.1)*	66	7 (10.6)*
LGA^a^	64	1 (1.6)	68	7 (10.3)	63	5 (7.9)	66	7 (10.6)

Data are *n*, means ± SD or medians (interquartile range) for continuous variables, and *n* (%) for categorical variables

^a^Excluding data for multiple pregnancies

^b^Based on IADPSG criteria

*p < 0.05 vs GBS

BMIM, BMI matched; FG, fasting plasma glucose level; G-60, plasma glucose level at 60 min after oral glucose load; G-120, plasma glucose level at 120 min after oral glucose load; GAD, gestational age at delivery; GBS, gastric bypass surgery patients; NW, normal weight; OB, obese controls; PE, pre-eclampsia

While the risk of pregnancy-induced hypertension or pre-eclampsia was lowest in patients with a history of gastric bypass surgery, no group-specific differences were found for obstetric outcome such as induction of labour, gestational age at delivery, or need of Caesarean section or neonatal intensive care. Of note, newborn infants of gastric bypass patients tended to be smaller with the highest risk of being SGA (electronic supplementary material Fig. 1). Maternal GDM status was not associated with increased birthweight percentiles (*p =* 0.901). Consequently, our conclusions remained the same after adjusting for GDM in multivariable analysis. Moreover, no interaction between a history of bariatric surgery and GDM status was found. Correlation analyses revealed that birthweight tended to be positively associated with fasting plasma glucose in women after gastric bypass (Spearman’s ρ = 0.29, *p =* 0.036), whereas this association was not observed for OGTT levels at 60 min or 120 min. Maternal hypoglycaemia at 120 min was not related to the risk of SGA offspring; however, the limited sample size has to be considered when drawing conclusions based on this finding.

## Discussion

Our results indicate altered glucose kinetics profiles during an OGTT, including a distinctive rise in plasma glucose levels at 60 min followed by hypoglycaemic episodes, in more than a half of pregnant patients with history of gastric bypass surgery. Moreover, the risk of pre-eclampsia or gestational hypertension was found to be reduced (gastric bypass vs obese mothers), whereas the newborn infants of mothers with a history of gastric bypass had a lower birthweight and were at a higher risk of being SGA compared with those of obese and normal weight controls. The differences in glucose kinetics and neonatal outcome appear to be independent of BMI because differences were also seen with BMI-matched patients.

The pathophysiological mechanisms leading to hypoglycaemia in gastric bypass patients are not fully understood. Although reports of OGTT data during pregnancy are sparse, studies in non-pregnant women suggest that altered gastric glucose transit followed by increased incretin peptide release and exaggerated insulin secretion from pancreatic beta cells, might provide an explanation [4]. Although we cannot provide further insight into these pathophysiological issues owing to our retrospective study design, these results have raised some important questions about screening and the definition of GDM. Recently, Johansson et al provided detailed information on pregnancy outcomes after bariatric surgery, including a lower risk of GDM (1.9% vs 6.8%) and LGA infants, although the risk of SGA was increased [5]. However, one criticism is that the use of routinely performed 2 h OGTT examinations in some previous studies might be considered inappropriate to detect hyperglycaemia [6]. Using the IADPSG definition, we observed a markedly increased incidence of GDM in bariatric surgery patients with glucose excursions, particularly those occurring at 60 min. While the clinical implication of this observation has not been established, it should be kept in mind that the GDM incidence was lowest in gastric bypass patients when fasting glucose and 120 min glucose levels (and not 60 min glucose levels) were used to classify hyperglycaemia in this subgroup. In addition, women with a history of gastric bypass showed lower fasting glucose levels, indicating some glycaemic improvement compared with obese, BMI-matched and even normal weight controls. These factors might contribute to the lower number of pregnant gastric bypass patients suffering from gestational hypertension and pre-eclampsia [7]. However, the specific impact of higher glycaemic variability on the course of pregnancy and pregnancy outcome, as well as the causes of the high incidence of SGA offspring, requires further investigation.

An unresolved question is how pregnancy-related hyperglycaemia should be screened in the growing population of gastric bypass women. Fasting glucose examination might serve as an acceptable marker to rule out GDM. Its lack in sensitivity might be improved by additionally including data on the patients’ medical history and sociodemographic variables [8]. Moreover, frequent capillary blood glucose examinations [9] or even continuous subcutaneous glucose monitoring [10] might represent diagnostic alternatives. These methods have the major advantage of detecting postprandial hyper- or hypoglycaemic episodes in real-life conditions (which can hardly be achieved by a single OGTT examination).

The retrospective nature of our study is a possible limitation because we were not able to provide information on nutritional status and its possible impact on pregnancy outcome or glycaemic variability. Although these issues need to be addressed in prospective investigations (including nutritional protocols and long-term glucose monitoring), our study represents a first attempt to describe OGTT alterations in pregnancies following gastric bypass surgery and has possible implications for GDM diagnosis. One advantage is that we could generate a large sample of OGTT glucose profiles to compare with lean, obese and weight-matched controls.

Based on our results, we conclude that plasma glucose concentrations after an oral glucose load are severely altered in pregnant gastric bypass patients. As the clinical implications of this observation are not yet clear, the diagnostic accuracy of the IADPSG criteria needs to be further examined for use in prospective longitudinal studies in this group of patients. Moreover, the potential risk of hypoglycaemia following an oral glucose load should be considered and alternative strategies should be discussed to rule out hyperglycaemia in this growing high-risk population.

## Electronic supplementary material

Below is the link to the electronic supplementary material.

ESM Fig. 1 (PDF 992 kb)

## Abbreviations

GDM: Gestational diabetes mellitus
IADPSG: International Association of Diabetes in Pregnancy Study Groups
LGA: Large for gestational age
SGA: Small for gestational age
