# Supplementary material for: Altered glucose profiles and risk for hypoglycaemia during oral glucose tolerance testing in pregnancies after gastric bypass surgery
Source: Diabetologia. 2016 Oct 18;60(1):153–7. doi: 10.1007/s00125-016-4128-8 (PMC6518072; doi:10.1007/s00125-016-4128-8)

ESM Figure 1: Newborn infant characteristics: A: birth weight percentiles (lines indicating 10th and 90th percentile); B: estimated mean difference and 95% family wise confidence levels of birth weight percentiles

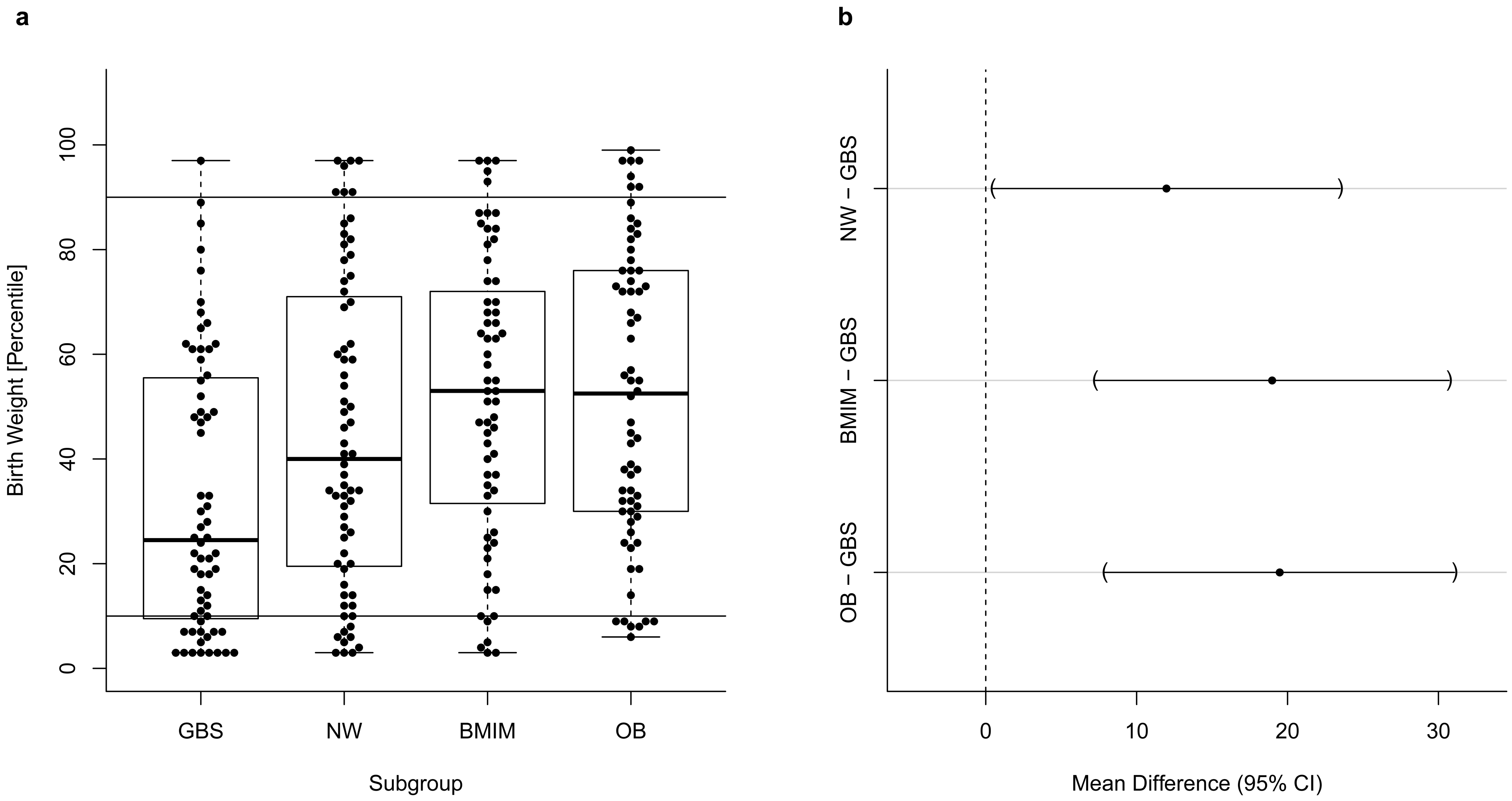

Supplement: Supplementary file 1 — (PDF 992 kb) [file 125_2016_4128_MOESM1_ESM.pdf]
